# Integrative phosphoproteome and interactome analysis of the role of Ubash3b in BCR-ABL signaling

**DOI:** 10.1038/s41375-019-0535-4

**Published:** 2019-08-09

**Authors:** Jevon A. Cutler, Savita Udainiya, Anil K. Madugundu, Santosh Renuse, Yaoyu Xu, Jaehun Jung, Kwang Pyo Kim, Xinyan Wu, Akhilesh Pandey

**Affiliations:** 1 McKusick-Nathans Institute of Genetic Medicine, Johns Hopkins University School of Medicine, Baltimore, MD 21205 USA; 2 Department of Laboratory Medicine and Pathology, Mayo Clinic, Rochester, MN 55905 USA; 3 Center for Molecular Medicine, National Institute of Mental Health and Neurosciences (NIMHANS), Hosur Road, Bangalore, Karnataka 560 029 India; 4 Institute of Bioinformatics, International Technology Park, Bangalore, Karnataka 560 066 India; 5 Manipal Academy of Higher Education (MAHE), Manipal, Karnataka 576104 India; 6 Department of Immunology, Institute of Basic Medical Sciences, Chinese Academy of Medical Sciences and School of Basic Medicine, Peking Union Medical College, State Key Laboratory of Medical Molecular Biology, 100005 Beijing, China; 7 Departments of Applied Chemistry, Institute of Natural Science, Global Center for Pharmaceutical Ingredient Materials, Kyung Hee University, Yongin, 17104 Republic of Korea; 8 Department of Biomedical Science and Technology, Kyung Hee Medical Science Research Institute, Kyung Hee University, Seoul, 02453 Republic of Korea; 9 Present Address: Department of Pediatric Oncology, Dana-Farber Cancer Institute, Harvard Medical School, Boston, MA 02210 USA

**Keywords:** Oncogenes, Biochemistry

## To the Editor

Chromosomal translocations involving the BCR and ABL genes result in the creation of the BCR-ABL oncogene that displays constitutive tyrosine kinase activity resulting in aberrant signaling leading to hematopoietic stem/progenitor cell transformation and leukemia. Recently, our laboratory reported a quantitative proteomics examination of signaling differences between the two main BCR-ABL variants, p190 and p210, which are associated with distinct leukemias in humans [1]. A similar study was reported by the Hantschel laboratory [2]. Although different methods were used to interrogate the BCR-ABL interactome, both groups identified ubiquitin-associated and Src-homology 3 (SH3) containing B (Ubash3b) as a major BCR-ABL interacting protein that showed increased interaction with p210 compared to p190.

**Fig. 1:**
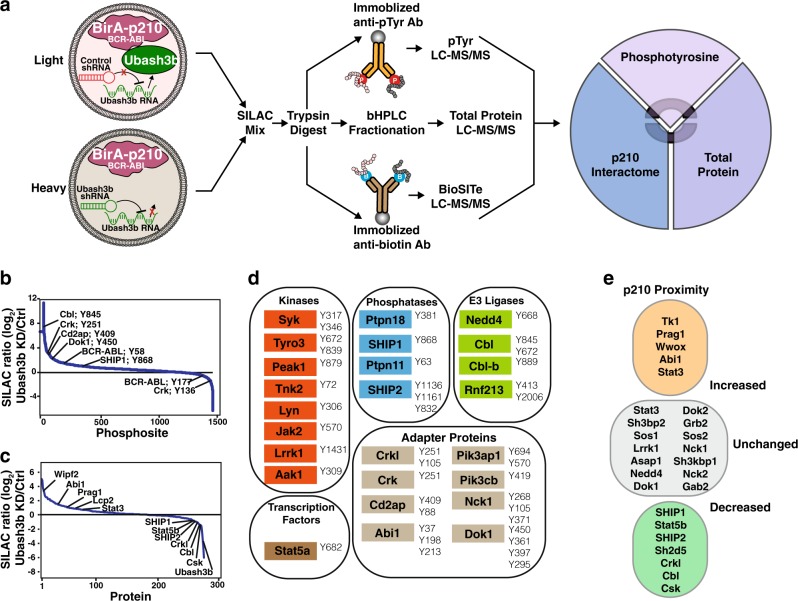
Integrated proteomic profiling of Ubash3b knockdown in p210 BCR-ABL cells. **a** The experimental workflow for integrated phosphotyrosine, total proteome, and interactome analysis. BaF3 cells harboring BirA*-p210-BCR-ABL upon Ubash3b KD (SILAC labeled: Light) and Luciferase KD (SILAC labeled: Heavy) were mixed in equal protein amounts and trypsin digested followed by immunoprecipitation by pan-phosphotyrosine antibody (phosphotyrosine analysis) and basic HPLC fractionation (total protein analysis) analyzed by LC-MS/MS. For BioSITe, SILAC labeled cells were treated with biotin and mixed in equal protein amount for trypsin digestion and anti-biotin antibody immunoprecipitation (p210 interactome) followed by LC-MS/MS analysis. **b** Waterfall plot for log_2_ fold-changes of tyrosine phosphorylated peptides in Ubash3b KD cells over control. **c** Relative abundance of biotinylated proteins by p210 BCR-ABL (log_2_ intensity ratio of Ubash3b KD/control cells). **d** Tyrosine hyperphosphorylated sites of proteins in Ubash3b KD compared to control and grouped into their molecular class. **e** p210 proximal proteins upon Ubash3b KD that increase, remain unchanged and decrease

Ubash3b, also known as suppressor of T-cell receptor signaling or Sts-1, is an ill-studied atypical tyrosine phosphatase with ubiquitin binding ability [3]. In our previous study, we hypothesized that Ubash3b plays an inhibitory role in BCR-ABL signaling through binding and dephosphorylating BCR-ABL and its interactors. The Hantschel lab recently solved the crystal structures of the p210 PH and DH domains, which are absent in the p190 variant, and demonstrated that loss-of-function mutations in the PH domain altered BCR-ABL localization, thereby reducing the interaction between Ubash3b and p210 [4]. Taken together, this suggests differential subcellular localization of Ubash3b as a mechanism by which it interacts more strongly with p210 as compared to p190. To better understand the global impact of Ubash3b on p210, its direct kinase substrates and proteins in its phosphotyrosine signaling network, we undertook an integrative approach by combining global phosphotyrosine profiling, proximity-dependent biotinylation (BioID) and total protein analysis to investigate p210 signaling upon Ubash3b knockdown (KD) (Fig. 1a). The BioID system was used to characterize Ubash3b function in p210 signaling by examining its interactome. Importantly, in all of our BioID experiments, we employed a new technique that we have recently developed, Biotinylation Site Identification Technology (BioSITe), which directly identifies biotinylated peptides thereby increasing the reliability of the identified interactors [5].

We previously described a system to analyze the signaling pathways of p210 by stable expression of BirA* tagged p210 BCR-ABL in Ba/F3 cells (Ba/F3 BirA*-p210; Supplementary Fig. S1) [1]. Here, we additionally used short hairpin RNA (shRNA) interference and generated Ubash3b knockdown (KD) and non-targeting control shRNA lines in Ba/F3 BirA*-p210 cells. We used the stable isotope labeling by amino acids in cell culture (SILAC) method [6] to achieve relative quantitation in our tandem mass spectrometry (LC-MS/MS) analysis. Ubash3b expression was reduced to >90% in the KD cells (Supplementary Fig. S1) and had a substantial effect on global tyrosine phosphorylation (Fig. 1b; Supplementary Fig. S1) and on the interactome of p210 (Fig. 1c). Of the 1421 unique tyrosine phosphorylation sites identified from 830 proteins, 379 sites (from 286 proteins) exhibited a substantial increase (≥2-fold) in tyrosine phosphorylation upon Ubash3b KD cells compared to control cells (Supplementary Table 1), representing many molecular classes (Fig. 1d). Many phosphorylation changes were confirmed by Western blot analysis (Supplementary Fig. S1). Ubash3b KD also revealed changes in the p210 interactome as measured by the BioID system. Using our BioSITe approach [5], combined with SILAC for the first time, we identified 260 biotinylated proteins of which 82 showed ≥1.5-fold increase and 20 showed ≤1.5-fold decrease upon Ubash3b KD (Fig. 1e; Supplementary Table 2). These results indicate that Ubash3b has a strong global impact by negatively regulating BCR-ABL protein interactions and signaling pathways. In addition, when we examined the effects of Ubash3b on the total proteome, we identified 7482 proteins with 201 proteins that were upregulated by ≥2-fold and 325 proteins downregulated by ≤2-fold (Supplementary Table 3).

To understand the functional impact of Ubash3b plays on p210 signaling, we first examined the phosphorylation status of p210 itself upon Ubash3b KD. As Ubash3b interacts with p210, it is likely that it could dephosphorylate p210 as Ubash3b is a tyrosine phosphatase. We observed 11 tyrosine residues in p210 to be hyperphosphorylated (>1.5-fold) upon Ubash3b KD (Supplementary Fig. S1; Supplementary Table 4) - this includes tyrosines important for BCR-ABL kinase activation, such as Y1314 (1.7-fold; Y412 in ABL1), located in the kinase domain, and Y58 (2.8-fold), located within the coiled-coil domain of ABL, which are involved in tetramerization/activation of BCR-ABL [7] and transformation potential [8]. Interestingly, Y177 was found to be hypophosphorylated (1.6-fold) upon Ubash3b KD possibly indicating more complex regulation independent of direct Ubash3b phosphatase activity. Y177 is critical in inducing disease in a mouse model of BCR-ABL-driven leukemia [9]. Nevertheless, the increased phosphorylation of several tyrosine residues on p210 suggests the elevation of kinase activity in the absence of Ubash3b or increase of tyrosine phosphorylation owing to lack of Ubash3b or both.

We next investigated the phosphorylation and changes in protein interactors of key molecules previously identified to be part of the p210 signaling complex. The E3 ubiquitin-protein ligase c-CBL (CBL), which has been shown to be required for BCR-ABL mediated transformation [10], was found to be strongly hyperphosphorylated at 2 sites, Y845 (100-fold) and Y672 (4.6-fold). Our interactome analysis showed that CBL also displayed a decrease in interaction with p210 (2.5-fold) (Supplemental Fig. S2) indicating that Ubash3b is likely mediating the p210/CBL interaction and potentially dephosphorylating both on tyrosines. CBL has been shown to coordinate with p210 to post-translationally regulate phosphatidylinositol 3,4,5-trisphosphate 5-phosphatase 1 (Inpp5d or SHIP1) leading to its degradation [11]. Ubash3b KD led to hyperphosphorylation of SHIP1 at Y868 (2.4-fold), along with a reduction of phosphorylation at Y43 (1.8-fold) and Y614 (2-fold) - all of these sites are functionally uncharacterized. The p210/SHIP1 interaction was decreased (1.6-fold) in Ubash3b KD indicating that Ubash3b plays a previously unappreciated role in the regulation of SHIP1. Similarly, phosphoinositide 3-kinase regulatory subunit 2 and inositol polyphosphate phosphatase-like 1 (Inppl1 or SHIP2) also showed differential regulation both in phosphorylation and interaction with p210 upon Ubash3b KD.

A number of essential BCR-ABL adapter proteins were also differentially phosphorylated upon Ubash3b KD, including Dok1, CrkL, Grb2, Gab2, Pik3ap, and CD2ap (Supplementary Table 1). The majority of these BCR-ABL associated adapter molecules did not display any differences in interaction with p210 except for CrkL (Fig. 1e; Supplementary Fig. S1) suggesting that adapter molecules are subject to Ubash3b phosphatase activity but these phosphosites may not be involved in regulating interaction with p210. Differential phosphorylation of Dok1 mediated by Ubash3b is interesting because it is known to bind to both SHIP1 and p210, shown to negatively regulate leukemogenic potential, act as a tumor suppressor and is a downstream target for ubiquitin-proteasome mediated downregulation in p210 mouse models [12].

Another molecule particularly important for BCR-ABL signaling found to be hyperphosphorylated and decreased in interaction with p210 upon Ubash3b KD was STAT5 (Supplementary Fig. S1). The transcription factor STAT5 is constitutively activated downstream of BCR-ABL and is essential for the establishment, maintenance, and even mediation of therapy resistance in BCR-ABL-positive leukemia [13]. Although BCR-ABL directly phosphorylates STAT5 [13], this transient interaction has only been detected in our sensitive BioID experiments [1, 5]. In our current data, upon Ubash3b KD, we observed hyperphosphorylation on several activating phosphosites, including Stat5a Y694 (1.9-fold), Y682 (3.8-fold), and Stat5b Y699 (1.8-fold), and hypophosphorylation of Y90 (1.4-fold) of Stat5a/b. The interaction of p210 to Stat5a was found to be decreased upon Ubash3b KD (Supplementary Fig. S1) suggesting that Ubash3b/p210/STAT5 interact with each other and Ubash3b dephosphorylates STAT5. These results indicate that Ubash3b has a profound impact on p210 signaling either directly by dephosphorylating STAT5 or indirectly by inhibiting p210 leading to diminished STAT5 activation.

**Fig. 2:**
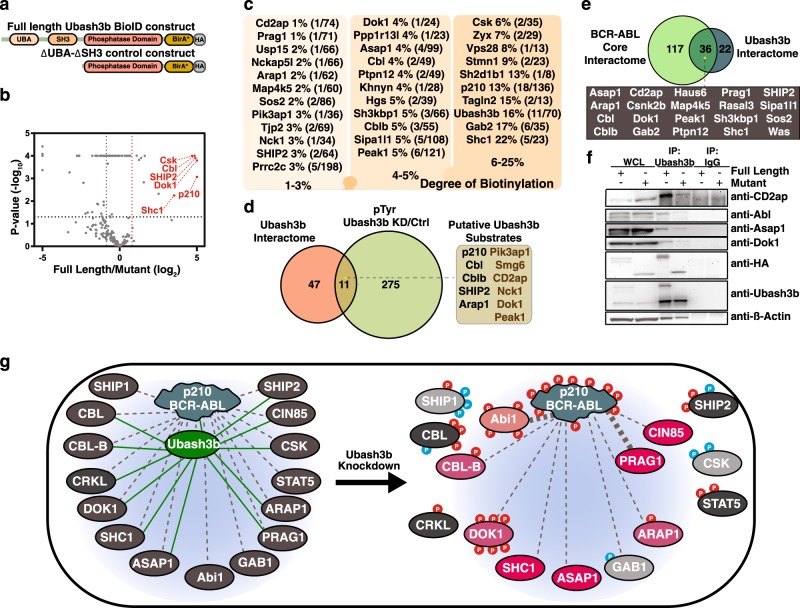
Interactome study of Ubash3b. **a** Domain structure of full-length and ∆UBA-∆SH3 deletion mutant with phosphatase domain Ubash3b constructs used for Ubash3b interactomes analysis. **b** Volcano plot of full-length vs. mutant Ubash3b BioID. **c** Ubash3b proximal proteins grouped by degree of biotinylation. **d** Venn diagram of overlap of tyrosine hyperphosphorylated proteins upon Ubash3b KD and Ubash3b interactomes. **e** Venn diagram showing common interactors of BCR-ABL and Ubash3b interactomes. **f** Western blot of indicated proteins after Ubash3b co-immunoprecipitation. **g** Model depicting p210 and Ubash3b interaction and tyrosine phosphorylation alterations upon Ubash3b KD. On the right, molecules involved in p210 signaling detected by our LC-MS/MS are shown (gray dotted lines) and Ubash3b (green lines). On the left, alterations of p210 interactors upon Ubash3b KD. Molecules with decreased or lost interactions are shown with no connecting lines and molecules with increased interaction with p210 are connected as thick dotted lines. Number of sites hyper and hypophosphorylated upon Ubash3b KD are shown in small bubbles in red and cyan respectively. Molecules in dark pink ovals are with no detected tyrosine phosphopeptides

To date, the interactome of Ubash3b has not been extensively investigated; however, limited investigation of Ubash3b in the context p210 signaling has been undertaken [14]. Understanding the identity of these interacting proteins should help identify potential substrates and common interacting proteins with p210. Thus, we next set out to investigate the Ubash3b interactome by again employing BioID with BioSITe. We designed constructs of C-terminal BirA* tagged full-length Ubash3b and a deletion mutant lacking the UBA and SH3 domains leaving only the phosphatase domain tethered to BirA* (Fig. 2a; Supplementary Fig. S2). Deletion of the UBA and SH3 domains should abolish most of its protein–protein interactions and serve as a control. Our LC-MS/MS analysis of the Ubash3b interactome resulted in the identification of 352 proteins, 42 of which were enriched ≥2-fold with full-length Ubash3b construct (Fig. 2b; Supplementary Table 5). We have recently suggested that the degree of biotinylation determined by BioSITe [5] can indicate stable and weak interactors (Fig. 2c). A comparative analysis of the core interactors of p210 from previous studies [1, 5, 14], and Ubash3b interactome from the current study revealed 36 proteins that interact with both p210 and Ubash3b (Fig. 2e). Many of the identified proteins overlapped with the previously mentioned proteins differentially interacting with p210 and phosphorylated, upon Ubash3b KD, including CBL, Crk, and SHIP2 which have been previously shown to interact with Ubash3b [14, 15]. Other identified interactors included NCK1, Shc1, Csk, Dok1, and Cd2ap (Fig. 2c). We also identified Arf-GAP with Rho-GAP domain, ANK repeat and PH domain-containing protein 1 (Arap1), SH3 domain-contain kinase-binding protein 1 (Sh3kbp1) also known as CBL interacting protein 85 kDa (CIN85), (Fig. 2c; Supplemental Table 5), which are known interactors of p210 [1, 4, 14]. Notably, we discovered two potentially novel interacting partners of both Ubash3b and p210, namely phosphoinositide 3-kinase adapter protein (Pik3ap1) and telomerase-binding protein EST1A (Smg6). We validated many of these interactions by co-immunoprecipitation studies (Fig. 2f; Supplemental Fig. S2). Putative substrates of Ubash3b were identified by overlapping the ratio of full-length Ubash3b over the mutant Ubash3b and the ratio of tyrosine hyperphosphorylation upon Ubash3b KD compared to control (Fig. 2d; Supplementary Table 1; Supplementary Table 5).

In summary, this study represents a novel multi-proteomic approach to dissect the role of Ubash3b in p210 BCR-ABL signaling. Ubash3b displays strong negative regulatory role exhibited by dephosphorylation of p210 and p210 signaling molecules in addition to its effects on proteins that interact with p210 (Fig. 2g). These data complement and are supported by findings from the study by Hantschel and colleagues in the accompanying paper that indicate a tumor-suppressive role of Ubash3b in p210-driven leukemia. These studies help define a critical role of Ubash3b in leukemia and should drive similar investigation in other malignancies driven by constitutively active tyrosine kinases.

## Supplementary information

Supplementary Figure Legends

Supplemental Methods

Supplementary Figure 1

Supplementary Figure 2

Supplementary Tables 1-5
