# Supplementary material for: Integrative phosphoproteome and interactome analysis of the role of Ubash3b in BCR-ABL signaling
Source: Leukemia. 2019 Aug 9;34(1):301–5. doi: 10.1038/s41375-019-0535-4 (PMC6934410; doi:10.1038/s41375-019-0535-4)
Supplement: Supplementary file 1 — Supplementary Figure Legends [file 41375_2019_535_MOESM1_ESM.docx]

**Supplementary Figure Legends**

**Fig. S1. Global phosphotyrosine analysis of BCR-ABL signaling upon Ubash3b KD. (a**) Domain structure of p210 BCR-ABL construct used for studying the role of Ubash3b in regulating BCR-ABL signaling. (**b**) Immunoblot of cell lysates from the stable cell lines generated to confirm the expression or KD of indicated proteins. (**c**) Immunoblot with 4G10-HRP of pan-phosphotyrosine 4G10 antibody immunoprecipitation for global tyrosine phosphorylation validation. (**d**) Tyrosine phosphorylated sites on the domain structure of p210 BCR-ABL. (**e**) Immunoblot of indicated proteins associated with BCR-ABL signaling for the whole cell lysate and phosphotyrosine immunoprecipitated p210 expressing upon Ubash3b KD.

**Fig. S2. BioSITe identified proteins as Ubash3b interactors and their validation by Co-Immunoprecipitation.** (**a**) Immunoblot of cell lysates from the stable cell lines generated to confirm the expression of full length and phosphatase domain Ubash3b. (**b**) Western blot of HA-Tag Ubash3b co-immunoprecipitated by SHC1 antibody. (**c**) Western blot of GAB2 co-immunoprecipitated by anti-HA Tag antibody.
