# Supplementary material for: Integrative phosphoproteome and interactome analysis of the role of Ubash3b in BCR-ABL signaling
Source: Leukemia. 2019 Aug 9;34(1):301–5. doi: 10.1038/s41375-019-0535-4 (PMC6934410; doi:10.1038/s41375-019-0535-4)
Supplement: Supplementary file 2 — Supplemental Methods [file 41375_2019_535_MOESM2_ESM.docx]

**Supplemental Methods**

**Plasmids, cloning, antibodies and reagents**

We cloned 5 shRNA hairpins designed by the Genetic Perturbation Platform (GPP) (https://portals.broadinstitute.org/gpp/public) targeting murine Ubash3b (TRCN0000099665, TRCN0000099667, TRCN0000099666, TRCN0000099668 and TRCN0000099669) into pLKO.1. TRC cloning vector was a gift from David Root (Addgene plasmid # 10878; <http://n2t.net/addgene:10878>; RRID:Addgene_10878) following the GPP instructions. We screened these 5 shRNA hairpins and a shRNA targeting luciferase in Ba/F3 cells stably expressing Myc-tagged-BirA*-p210-BCR-ABL which revealed TRCN0000099668 to be the most efficient hairpin to knockdown Ubash3b. Full length Ubash3b and deletion mutant lacking UBA and SH3 domains consisting of the phosphatase domain tethered to C-terminal BirA* HA tag were synthesized and cloned into the pBABE-puro vector by GenScript Biotech Corp, New Jersey, USA.

Antibodies used for this study include: anti-ABL1 (emdMillipore, Burlington, MA, #MABT203), anti-Ubash3b/STS-1 (Santa Cruz Biotechnology (SCB), Dallas, TX sc-514612), anti-Ubash3b/STS-1 (Abcam, Cambridge, MA, #34781), anti-Biotin (Bethyl Laboratories, Montgomery, TX, #A150-109A), anti-Rabbit IgG Isotype control (Cell Signaling Technology (CST), Danvers, MA, #3900), anti-SHIP1 (CST #2728), anti-Gab2 (emdMillipore #06-967), anti-Stat5a (SCB. sc-1081), anti-DOK1 (Santa Cruz Biotech. sc-6929), anti-Cbl (7G10) (emdMillipore #05-440), anti-Csk (SCB, sc-286), anti-CrkL (Santa Cruz Biotech. sc-319), anti-CD2ap (Santa Cruz Biotech. sc-9137), anti-ASAP1 (SCB. sc-136392), anti-SHC (BD Biosciences, San Jose, CA, #610081), anti-HA tag (Abcam #ab9110), anti-phosphotyrosine 4G10® Platinum HRP Conjugate (emdMillipore #16-105), and anti-β-Actin (CST #4970). Heavy amino acids for SILAC were purchased from Cambridge Isotopes, Tewksbury, MA; heavy (lysine-^13^C_6_, ^15^N_2_ #CNLM-291, arginine-^13^C_6_, ^15^N_4_ #CNLM-539) labels were dissolved in arginine- and lysine-deficient RPMI 1640 medium (ThermoFisher, Waltham, MA, #89984) and then sterile filtered.

**Cell culture and experimental set up**

All cells were cultured in RPMI 1640 supplemented with 10% fetal bovine serum. Luciferase KD BirA*-p210-BCR-ABL expressing Ba/F3 cells were labelled with heavy ^13^C_6_, ^15^N_2_-Lys and ^13^C_6_, ^15^N_4_-Arg and Ubash3b KD BirA*-p210-BCR-ABL expressing cells were labelled with light SILAC medium. Separate replicate cultures were established for each experiment; three independent replicates were used for the phosphotyrosine experiments, two replicates for the Ubash3b KD p210 BioID with BioSITe experiment, 2 replicates for total proteome experiment, and, three replicates were used for label-free Ubash3b BioID with BioSITe experiment. Cells used for BioID experiments were treated with 50 μM of biotin for 24 hours. Cells were harvested, washed with PBS in large volume washes and pelleted for peptide enrichment preparation.

**Peptide enrichment preparation**

Protein extraction was carried out by sonication (three rounds, duty cycle 30%, 20 s pulses) in 50 mM TEABC and 8 M urea. The protein concentration of samples was measured by BCA assay. For SILAC experiments, heavy and light samples were mixed 1:1 and a total of 10 mg of lysate per replicate was then reduced and alkylated were by serial incubation of 10 mM DTT for 30 min and by 20 mM IAA for 30 min in the dark. Lysate was diluted to 2 M urea by adding three cell lysate volumes of 50 mM TEABC. The proteins were digested with trypsin (1:20 of trypsin to protein) at 37 °C overnight. The resulting tryptic peptides were desalted using a Sep-PAK C_18_ column and subsequently lyophilized.

**Immunoaffinity purification of phosphotyrosine peptides and total protein analysis**

Peptides were resuspended in IAP buffer (CST #9993) and pH was brought to neutral with 1M tris base. Phosphopeptides were enriched using the PTMScan® Phospho-Tyrosine Mouse mAb (P-Tyr-100) Kit (CST #5636) and anti-phosphotyrosine immunoprecipitation was carried out following the manufacturer’s instructions. For total protein analysis, a 4 mg aliquot of SILAC mix was also subjected to a separate in-solution tryptic digestion and was fractionated by basic-pH reversed-phase liquid chromatography into 24 fractions which were dried, desalted, and concentrated using in-house prepared C_18_ reversed-phase column.

**BioID with BioSITe**

Protein G agarose beads (Millipore Sigma, #16-266) were washed twice with PBS and 100 *μ*g of anti-biotin antibody (Bethyl Laboratories, Inc. A150-109A) were coupled to 120 *μ*L of protein G bead slurry, pre-replicate, overnight at 4 °C. Antibody-coupled beads were further washed with PBS once and BioSITe capture buffer (50 mM Tris-HCL, 150 mM NaCl, 0.5% Triton X-100) twice. Lyophilized peptides were dissolved in 1 mL of BioSITe capture buffer and pH solution was adjusted to neutral (7.0 to 7.5). Peptides were subsequently incubated with anti-biotin antibody-bound protein G beads for 1 h at 4 °C. The bead slurry was sequentially washed three times with BioSITe capture buffer, three times with 50 mL of Tris-HCL, and two times with ultrapure water. Biotinylated peptides were eluted with four rounds of 200 *μ*L elution buffer (80% acetonitrile and 0.2% trifluoroacetic acid in water). The eluents were dried, desalted, and concentrated using homemade C_18_ reversed-phase column.

**Liquid chromatography tandem mass spectrometry analysis:**

Phosphotyrosine-enriched peptide samples were reconstituted in 10 µL of Solvent A (0.1% Formic Acid in water) and 8 µL of each sample was analyzed by nano-LC-MS/MS using Orbitrap Fusion Lumos (Thermo Scientific, San Jose) interfaced with Ultimate 3000 RSLCnano UPLC system (Thermo Scientific). Peptides were loaded on a pre-column (100 μm id × 5 mm, C_18_ PepMap100, 5 μm, Thermo Scientific) at 8 μL/min for 6 minutes using 0.1% Formic acid in water. After 6 min, the peptides were separated on an EasySpray column (75 μm id × 50 cm, C_18_ PepMap, 2 μm, Thermo Scientific) at 300 nL/min using solvent B (0.1% formic Acid in 95% Acetonitrile). The gradient consisted of initial step of 8–32% B over 100 min followed by 32–50% over 7 min, 32–90% B over 3 min, held at 90%B for 5 min and then equilibrated for 20 min at 5% B, where mobile phase A consisted on water containing 0.1% formic acid and mobile phase B consisted of 95/5 acetonitrile/water containing 0.1% formic acid. Separation was performed at 45°C and the total acquisition time was 140 min. The mass spectrometer was fitted with an EasySpray source (Thermo Scientific) and operated in DDA manner. Each DDA cycle consisted of one OT MS survey scan acquired at 120,000 resolution at m/z 200 and precursors ions meeting user defined criteria such as charge state, monoisotopic precursor selection, intensity, and dynamic exclusion were selected for MS2 based on “cycle time” of 3 sec. Precursor ions were isolated using the quadrupole (1.4 Th isolation width with isolation offset of 0.4 Th) and fragmentation was carried out using higher-energy dissociation (HCD) method using normalized collision energy (NCE) of 32. The MS2 scans were detected in the orbitrap at 30,000 resolution at m/z 200.

**Data analysis**

Proteome Discoverer (v 2.2; Thermo Scientific) suite was used for quantitation and identification of peptides from LC–MS/MS runs. Spectrum selector was used to import spectrum from raw file. During MS/MS preprocessing, the top 10 peaks in each window of 100 m/z were selected for database search. The tandem mass spectrometry data were then searched using SEQUEST algorithm against protein databases (mouse NCBI RefSeq 73 (58039 entries) with the addition of fasta file entries; BirA*-p210-BCR-ABL (for p210 BioID with BioSITe/pTyr/total protein experiment), Ubash3b-BirA* and p210-BCR-ABL (For Ubash3b BioID with BioSITe experiment) and common contaminant proteins. The search parameters for identification of biotinylated peptides were as follows: (a) trypsin as a proteolytic enzyme (with up to three missed cleavages); (b) peptide mass error tolerance of 10 ppm; (c) fragment mass error tolerance of 0.02 Da; and (d) carbamido-methylation of cysteine (+57.02146 Da) as a fixed modification and oxidation of methionine (+15.99492 Da) and biotinylation of lysine (+226.07759 Da) as variable modifications. In case of SILAC BioSITe experiment, custom variable modification of biotinylated heavy lysine (+234.091789 Da) was added. As the routine proteomics workflows fail to quantify variable custom modifications under SILAC configurations, we used PyQuant^1^ for quantification of biotinylated peptides. Peptides and proteins were filtered at a 1% false-discovery rate (FDR) at the PSM level using percolator node and at the protein level using protein FDR validator node, respectively. For SILAC experiments relative quantification of identified peptides was carried out using PyQuant by deriving peak areas of light and heavy isotopic peptides from MS1 level data in raw files. Then peak areas of identical peptides were merged to derive overall abundance estimates of samples being compared. Site level information of biotinylated peptides was then processed to derive abundance and degree of biotinylation at protein level.

For Ubash3b BioID experiment MS1 level quantification was carried out with the Minora Feature Detector, using the program’s standard parameters and all of the raw files from the three replicates were quantified together. Unique and razor peptides both were used for peptide quantification, while protein groups were considered for peptide uniqueness. Identified protein and peptide spectral match (PSM) level data were exported as tabular files from Proteome Discoverer 2.2. We used an in-house Python script to compile the peptide level site information mapped to RefSeq databases. We eliminated all non-biotinylated peptides from our analysis. The summary count on the number of supported peptides, PSMs, number of biotinylation sites and quantification was then calculated at the protein level.

**Co-Immunoprecipitation and phosphotyrosine immunoprecipitation**

Cells were gently lysed in co-IP buffer (20 mM Tris HCl, 100 mM NaCl, 1% NP-40 and 2.5 mM EDTA) for 1 hour rotation at 4^o^C and centrifuged at 13000 rpm for 10 min. Protein from the supernatant was estimated using BCA kit. 3 mg of lysate was incubated with 6 μg of antibody and rotated for 3 hour at 4^o^C. 50 μl of Protein G Dynabeads (Thermo Scientific #10003D) were washed three times in co-IP buffer and added to the lysate and antibody mix and further rotated for 1 hour at 4^o^C followed by 4 times washing with co-IP buffer and the co-precipitating proteins attached with magnetic beads were denatured with 2X Laemmli buffer (Bio-Rad #1610747). Whole eluate was used for Western blot.

Phosphotyrosine immunoprecipitation was performed by incubating 1mg of lysate with 20 μl or 4 mg of lysate in mRIPA with 100 μl of 3 times washed α-phosphotyrosine 4G10®, agarose conjugate (emdMillipore #16-199) for overnight rotation at 4^o^C. Next day the beads were washed 3 times with mRIPA buffer and the pulled down proteins were denatured with 4X LDS dye and the whole eluate was loaded on to the SDS-PAGE gels for Western blot. For the indicated antibodies, the blot was developed using ECL chemiluminescence.

**Data Availability**

All raw mass spectrometry (MS) files used in this experiment have been uploaded to the ProteomeXchange Consortium (http://proteomecentral.proteomexchange.org) via the PRIDE partner repository.

**Experimental Design and Statistics**

Phosphotyrosine analysis and Ubash3b BioID with BioSITe experiments were performed in triplicate. Total proteome and p210 BioSITe experiments were performed in duplicate. Co-immunoprecipitation and phosphoprotein immunoprecipitation experiments were performed in two independent experiments. Two tailed Student’s t-test (independent samples) was performed with the null hypothesis (H_0_) of no difference in means in the sample groups compared to calculate probabilities (p-value). Fold-changes between groups were derived by taking ratio of sample means from triplicate measurements.

**Supplemental Methods References**

1 Mitchell CJ, Kim M-S, Na CH, Pandey A. PyQuant: A Versatile Framework for Analysis of Quantitative Mass Spectrometry Data. *Mol Cell Proteomics* 2016; **15**: 2829–2838.
