# Supplementary figures and images for: Integrative phosphoproteome and interactome analysis of the role of Ubash3b in BCR-ABL signaling
Source: Leukemia. 2019 Aug 9;34(1):301–5. doi: 10.1038/s41375-019-0535-4 (PMC6934410; doi:10.1038/s41375-019-0535-4)

# Supplementary Figure 1

**a**

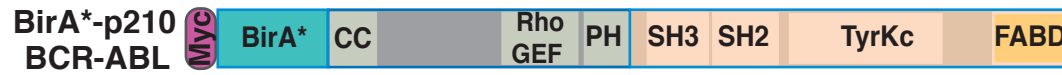

**b**

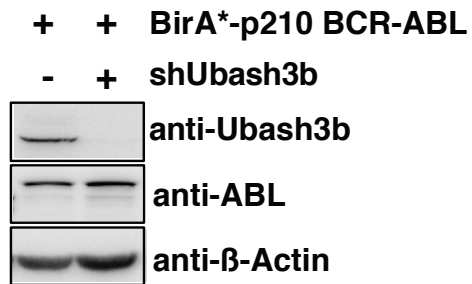

**c**

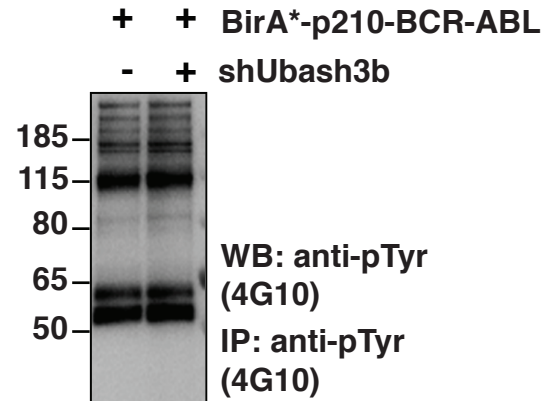

**d**

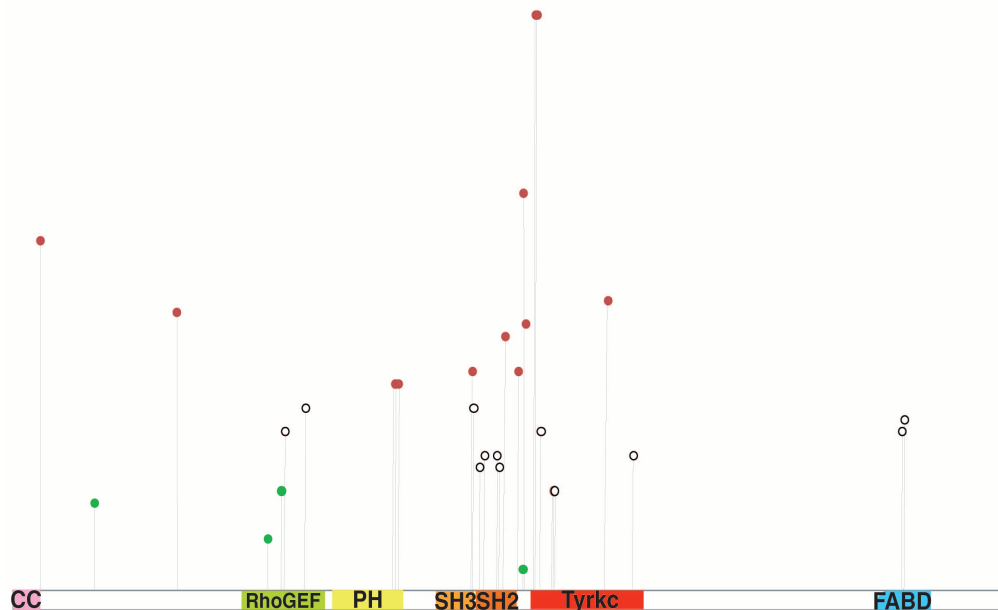

**e**

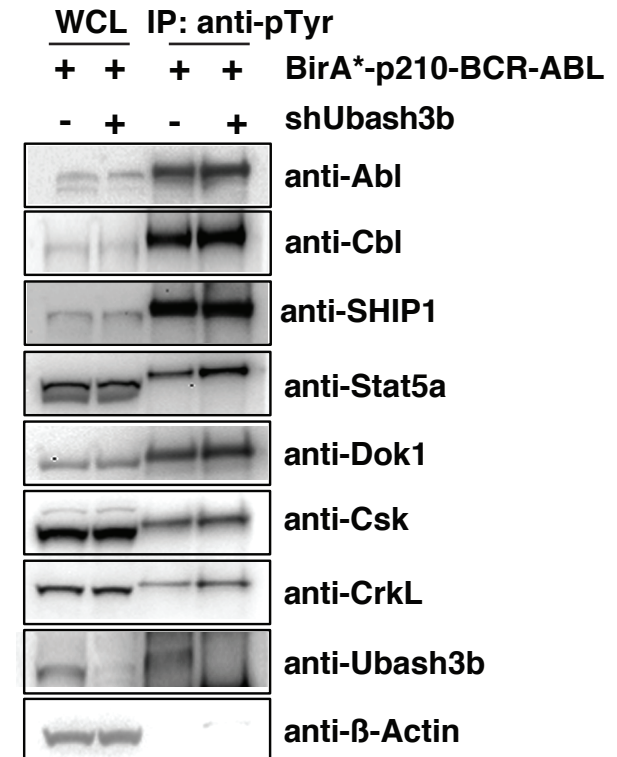

Supplement: Supplementary file 3 — Supplementary Figure 1 [file 41375_2019_535_MOESM3_ESM.pdf]

# Supplementary Figure 2

**a**

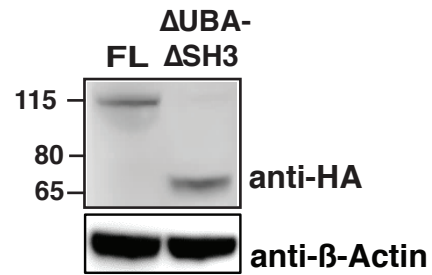

**b**

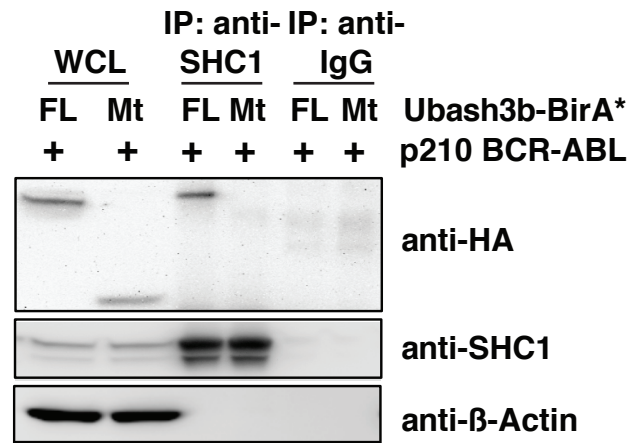

**c**

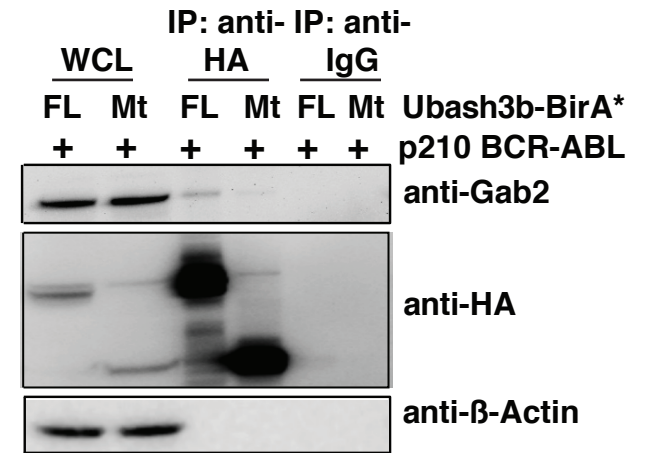

Supplement: Supplementary file 4 — Supplementary Figure 2 [file 41375_2019_535_MOESM4_ESM.pdf]
